# Supplemental Smartamine M in higher-energy diets during the prepartal period improves hepatic biomarkers of health and oxidative status in Holstein cows

**DOI:** 10.1186/s40104-017-0147-7

**Published:** 2017-02-06

**Authors:** Mario Vailati-Riboni, Johan S. Osorio, Erminio Trevisi, Daniel Luchini, Juan J. Loor

**Affiliations:** 1 Mammalian NutriPhysioGenomics, Department of Animal Sciences and Division of Nutritional Sciences, University of Illinois, Urbana, IL 61801 USA; 2 Istituto di Zootecnica Facoltà di Scienze Agrarie, Alimentari e Ambientali, Università Cattolica del Sacro Cuore, 29122 Piacenza, Italy; 3 Adisseo NA, Alpharetta, GA 30022 USA; 4 Dairy and Food Science Department, South Dakota State University, 1111 College Ave, 113H Alfred DairyScience Hall, Brookings, SD 57007 USA

**Keywords:** Energy, Methionine, Nutrigenomics, Transition period

## Abstract

**Background:**

Feeding higher-energy prepartum is a common practice in the dairy industry. However, recent data underscore how it could reduce performance, deepen negative energy balance, and augment inflammation and oxidative stress in fresh cows. We tested the effectiveness of rumen-protected methionine in preventing the negative effect of feeding a higher-energy prepartum. Multiparous Holstein cows were fed a control lower-energy diet (CON, 1.24 Mcal/kg DM; high-straw) during the whole dry period (~50 d), or were switched to a higher-energy (OVE, 1.54 Mcal/kg DM), or OVE plus Smartamine M (OVE + SM; Adisseo NA) during the last 21 d before calving. Afterwards cows received the same lactation diet (1.75 Mcal/kg DM). Smartamine M was top-dressed on the OVE diet (0.07% of DM) from -21 through 30 d in milk (DIM). Liver samples were obtained via percutaneous biopsy at -10, 7 and 21 DIM. Expression of genes associated with energy and lipid metabolism, hepatokines, methionine cycle, antioxidant capacity and inflammation was measured.

**Results:**

Postpartal dry matter intake, milk yield, and energy-corrected milk were higher in CON and OVE + SM compared with OVE. Furthermore, milk protein and fat percentages were greater in OVE + SM compared with CON and OVE. Expression of the gluconeogenic gene *PCK1* and the lipid-metabolism transcription regulator *PPARA* was again greater with CON and OVE + SM compared with OVE. Expression of the lipoprotein synthesis enzyme *MTTP* was lower in OVE + SM than CON or OVE. Similarly, the hepatokine *FGF21*, which correlates with severity of negative energy balance, was increased postpartum only in OVE compared to the other two groups. These results indicate greater liver metabolism and functions to support a greater production in OVE + SM. At 7 DIM, the enzyme *GSR* involved in the synthesis of glutathione tended to be upregulated in OVE than CON-fed cows, suggesting a greater antioxidant demand in overfed cows. Feeding OVE + SM resulted in lower similar expression of *GSR* compared with CON. Expression of the methionine cycle enzymes *SAHH* and *MTR*, both of which help synthesize methionine endogenously, was greater prepartum in OVE + SM compared with both CON and OVE, and at 7 DIM for CON and OVE + SM compared with OVE, suggesting greater Met availability. It is noteworthy that *DNMT3A*, which utilizes S-adenosylmethionine generated in the methionine cycle, was greater in OVE and OVE + SM indicating higher-energy diets might enhance DNA methylation, thus, Met utilization.

**Conclusions:**

Data indicate that supplemental Smartamine M was able to compensate for the negative effect of prepartal energy-overfeeding by alleviating the demand for intracellular antioxidants, thus, contributing to the increase in production. Moreover Smartamine M improved hepatic lipid and glucose metabolism, leading to greater liver function and better overall health.

**Electronic supplementary material:**

The online version of this article (doi:10.1186/s40104-017-0147-7) contains supplementary material, which is available to authorized users.

## Background

The transition period, defined as last 3 weeks prepartum through 3 weeks postpartum, is one of the most important stages of lactation in dairy cattle. Years of strong genetic selection and improvement have allowed modern dairy cows to reach high production performance, both in quantity and quality. However, this has made the transition between late pregnancy to early lactation a significant period of metabolic and immune challenges [1–3]. Because failure to adequately meet these challenges can compromise production, induce metabolic diseases, and increase rates of culling in early lactation [4], the management of the transition cow remains a focal point for dairy producers.

Following the “steaming up” concept of RB Boutflour [5], transition cows during the dry period were first traditionally offered a high fiber/low energy density ration, to then increase the energy density of the ration with a lower fiber content in the last month of gestation (i.e. “close-up” period). This early century practice is still embedded in the modern dairy industry. However, multiple studies have consistently reported negative effects of prepartum energy overfeeding on cow health and productivity. Among these, prepartum hyperglycemia and hyperinsulinemia together with marked postpartum adipose tissue mobilization (i.e., greater blood NEFA concentration) [6–11] have strong negative impact on postpartal health indices [12–15].

Our general hypothesis was that supplementation with rumen-protected methionine (Smartamine M, Adisseo NA) could ameliorate the transition to lactation and the health status of the cows, while controlling and reducing the negative effects of prepartal excess energy. In fact, methionine (Met) itself was able to increase both quantity and quality of production [16, 17], controlling the inflammatory and the oxidative stress status that characterize the transition period [18–20]. These outcomes are partly due to Met’s ability to enhance liver function, reducing triacylglycerol accumulation and improving the metabolic capacity of the liver to orchestrate the metabolic transition into lactation [16–20]. Furthermore, Met itself, and several of its metabolites, display an immunonutritional role both in humans [21–24] and in dairy cows [16]. Therefore, in the present study we used serum and plasma biomarkers coupled with targeted hepatic transcriptome analysis from transition cows fed prepartum either a control low energy, a higher-energy, or a higher-energy diet supplemented with rumen-protected Met. Production and immune responses have been published elsewhere [25].

## Methods

### Experimental design and dietary treatments

All procedures were approved by the Institutional Animal Care and Use Committee (IACUC) of the University of Illinois. Complete details of the experimental design and animal management have been reported previously [25]. Briefly, 65 multiparous Holstein were enrolled and completed the trail remaining healthy throughout the length of the study. All cows were fed ad libitum the same control lower-energy diet (CON; NE_L_ = 1.24 Mcal/kg DM; no Met supplementation) during the far-off dry period (i.e., -50 to -21 d relative to parturition). Consequently, during the close-up period (i.e. -21 d to calving), cows were randomly allocated to either a higher-energy diet (OVE; NE_L_ = 1.54 Mcal/kg DM), OVE plus Smartamine M (OVE + SM; Adisseo, NA) or remained on CON. The same basal lactation diet (NEL = 1.75 Mcal/kg DM) was fed to all cows postpartum until d 30 relative to parturition. Smartamine M was top-dressed during the entire experiment over the OVE or lactation diet from -21 through 30 d relative to parturition at a rate of 0.07% of offered DM. For the current study, only a subset of cows were considered for blood biomarker (*n* = 10 per group) and hepatic gene expression (*n* = 8 per group) analyses.

### Blood sampling and biomarker analysis

Blood was sampled at -26, -21, -10, 7, 14 and 21 d relative to parturition by coccygeal venipuncture using evacuated tubes (BD Vacutainer; BD and Co., Franklin Lakes, NJ) containing either clot activator or lithium heparin for serum and plasma, respectively. Blood was used for determination of (i) metabolic biomarkers: cholesterol, creatinine, growth hormone (GH), insulin-like growth factor 1 (IGF-1), leptin, urea; (ii) liver health biomarkers: albumin, bilirubin, ceruloplasmin, gamma-glutamyl-transpeptidase (GGT), glutamic oxaloacetic transaminase (GOT), haptoglobin, interleukin 6, serum amyloid A (SAA); (iii) and oxidative status biomarkers: β-carotene, glutathione, nitric oxides (NO_x_, NO_2_, NO_3_), paraoxonase, antioxidant capacity (oxygen radical absorbance capacity, ORAC), total reactive oxygen metabolites (ROM), tocopherol.

Concentration of albumin, cholesterol, bilirubin, creatinine, urea, GOT, and GGT were assessed using kits purchased from Instrumentation Laboratory (Lexington, MA) using a clinical auto-analyzer (ILAB 600, Instrumentation Laboratory). Concentrations of ROM were analyzed with the d-ROMs-test, purchased from Diacron (Grosseto, Italy). Concentrations of haptoglobin, ceruloplasmin, paraoxonase and NOx were analyzed using the methods previously described [26–28], adapting the procedures to a clinical auto-analyzer (ILAB 600, Instrumentation Laboratory). SAA and ORAC determinations were performed using the Synergy 2 Multi-Detection Microplate Reader (BioTek Instruments, Inc., Winooski, VT). SAA concentration was assessed with a commercial ELISA immunoassay kit (Tridelta Development Ltd., Maynooth, Co. Kildare, Ireland), while ORAC was determined measuring the fluorescent signal from a probe (fluorescein) that decreases in the presence of radical damage [29]. Quantification of GH, IGF-1, and leptin concentration was as previously described [14]. Bovine IL-6 (Cat. No. ESS0029; Thermo Scientific, Rockford, IL) plasma concentration was determined using commercial ELISA kits, while plasma vitamin A, vitamin E, and β-carotene were extracted with hexane and analyzed by reverse- phase HPLC using an Allsphere ODS-2 column (3 μm, 150 × 4.6 mm; Grace Davison Discovery Sciences, Deerfield, IL), a UV detector set at 325 nm (for vitamin A), 290 nm (for vitamin E), or 460 nm (for β-carotene), and 80:20 methanol:tetrahydrofurane as the mobile phase.

### Hepatic gene expression analysis

Liver tissue was harvested via percutaneous biopsy under local anesthesia at -10, 7 and 21 d relative to parturition. Tissue samples were immediately snap frozen in liquid nitrogen and then stored at -80 °C. Complete information about RNA extraction and qPCR procedures can be found in Additional file 1. Briefly, RNA samples were extracted from the frozen tissue and used for cDNA synthesis using established protocols in our laboratory [30]. The qPCR performed was SYBR Green-based, using a 6-point standard curve. Genes selected for transcript profiling are associated with (i) energy metabolism: insulin like growth factor-1 (*IGF1*), pyruvate carboxylase (*PC*), phosphoenolpyruvate carboxykinase 1 (*PCK1*), pyruvate dehydrogenase kinase 4 (*PDK4*); (ii) fatty acid metabolism: acyl-CoA oxidase 1 (*ACOX1*), apolipoprotein B (*APOB*), γ-butyrobetaine hydroxylase 1 (*BBOX1*), carnitine palmitoyltransferase 1A (*CPT1A*), 3-hydroxy-3-methylglutaryl-CoA synthase 2 (*HMGCS2*), microsomal triglyceride transfer protein (*MTTP*), peroxisome proliferator activated receptor α (*PPARA*), solute carrier family 22 member 5 (*SLC22A5*), trimethyllysine hydroxylase, ε (*TMLHE*); (iii) hepatokines: angiopoietin like 4 (*ANGPTL4*), fibroblast growth factor 21 (*FGF21*); (iv) the methionine cycle: betaine--homocysteine S-methyltransferase (*BHMT*), betaine--homocysteine S-methyltransferase 2 (*BHMT2*), DNA (cytosine-5-)-methyltransferase 1 (*DNMT1*), DNA (cytosine-5-)-methyltransferase 3 α (*DNMT3A*), methionine adenosyltransferase 1A (*MAT1A*), 5-methyltetrahydrofolate-homocysteine methyltransferase (*MTR*), phosphatidylethanolamine N-methyltransferase (*PEMT*), S-adenosylhomocysteine hydrolase (*SAHH*); (v) the antioxidant system: cystathionine-beta-synthase (*CBS*), cysteine sulfinic acid decarboxylase (*CSAD*), cystathionine gamma-lyase (*CTH*), glutamate-cysteine ligase catalytic subunit (*GCLC*), glutathione peroxidase 1 (*GPX1*), glutathione reductase (*GSR*), glutathione synthetase (*GSS*), superoxide dismutase 1, soluble (*SOD1*), superoxide dismutase 2, mitochondrial (*SOD2*); (vi) and the inflammatory response: ceruloplasmin (*CP*), haptoglobin (*HP*), nuclear factor κB subunit 1 (*NFKB1*), retinoid X receptor α (*RXRA*), serum amyloid A2 (*SAA2*), suppressor of cytokine signaling 2 (*SOCS2*), signal transducer and activator of transcription 3 (*STAT3*), signal transducer and activator of transcription 5B (*STAT5B*). Primer sequences and qPCR performances are reported in Additional file 1.

### Statistical analysis

**Fig. 1:**
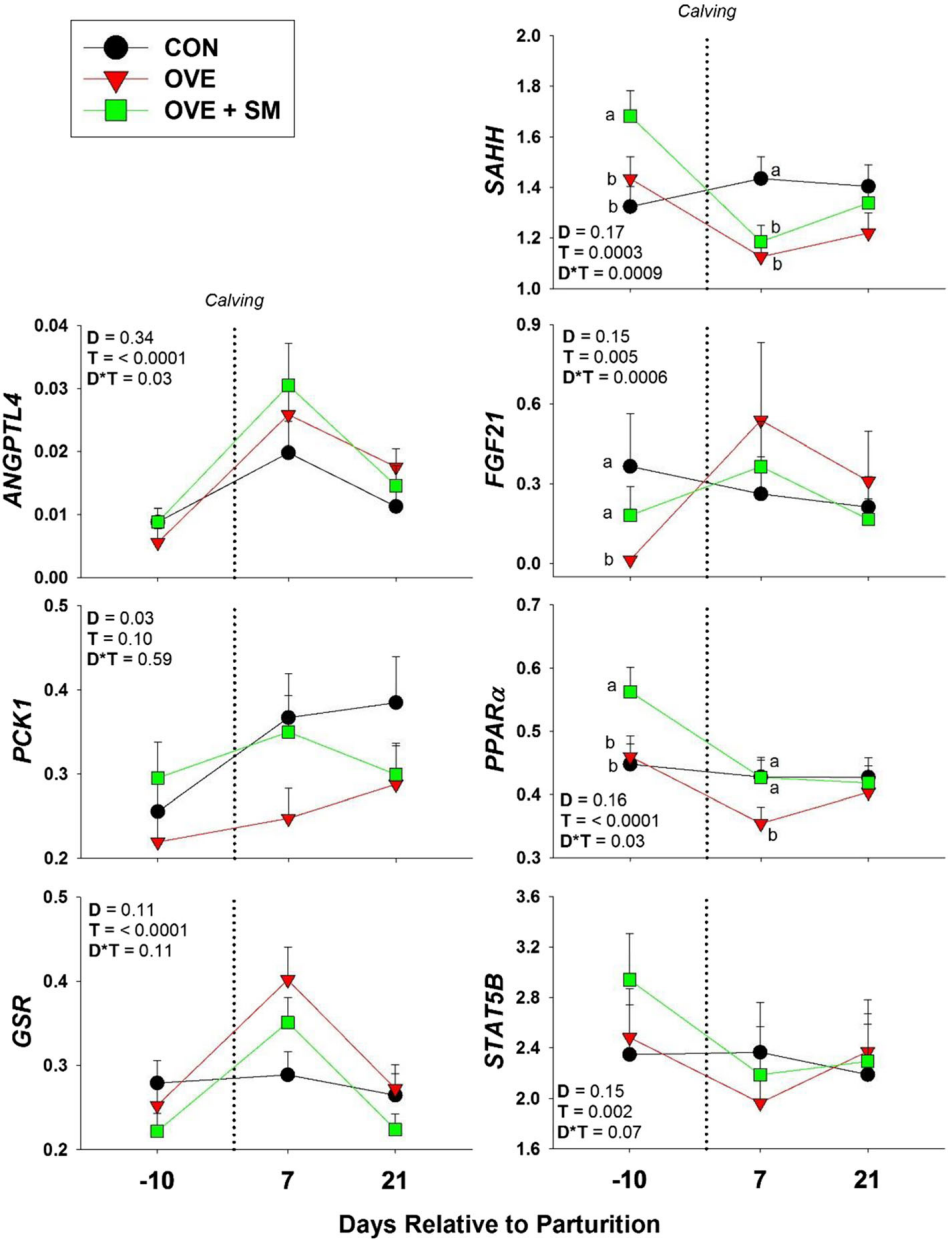
Effect of feeding a control lower-energy diet (CON, 1.24 Mcal/kg DM; high-straw) during the whole dry period (~50 d), a higher-energy (1.54 Mcal/kg DM) diet without (OVE) rumen-protected methionine during the last 21 d before calving, or OVE plus rumen-protected methionine (Smartamine M; OVE + SM; Adisseo NA) from -21 d before calving through the first 30 d postpartum on hepatic gene expression (log_2_ back-transformed LSM) in Holstein cows

After normalization with the geometric mean of the internal control genes, qPCR data were log_2_ transformed prior to statistical analysis to obtain a normal distribution. Statistical analysis was performed with SAS (v9.3). Both datasets (blood and qPCR) were subjected to ANOVA and analyzed using repeated measures ANOVA with PROC MIXED. The statistical model included diet (D; CON, OVE, and OVE + SM), time (T; d -26, -21, -10, 7, 14, and 21 for blood biomarkers, d -10, 7, and 21 for qPCR analysis) and their interaction (D*T) as fixed effect. Cow, nested within treatment, was the random effect. For blood data, data pre-treatment at d-26 relative to parturition, when available, were used as a covariate. The Kenward-Roger statement was used for computing the denominator degrees of freedom, while spatial power was used as the covariance structure. Data were considered significant at a *P* ≤ 0.05 using the PDIFF statement in SAS. For ease of interpretation, expression data reported in Table 1 and Fig. 1 are the log_2_ back-transformed LSM that resulted from the statistical analysis. Standard errors were also adequately back-transformed.

**Table 1:** Effect of feeding a control lower-energy diet (CON, 1.24 Mcal/kg DM; high-straw) during the whole dry period (~50 d), a higher-energy (1.54 Mcal/kg DM) diet without (OVE) rumen-protected methionine during the last 21 d before calving, or OVE plus rumen-protected methionine (Smartamine M; OVE + SM; Adisseo NA) from -21 d before calving through the first 30 d postpartum on hepatic gene expression (relative mRNA abundance, log_2_ back-transformed LSM) in Holstein cows

	Diet^1^				*P*-value^3^		
	CON	OVE	OVE + SM	SE^2^	D	T	D*T
Energy metabolism							
*IGF1*	1.91	1.97	2.41	0.22	0.19	<.0001	0.17
*PC*	0.24	0.23	0.20	0.02	0.24	<.0001	0.09
*PCK1*	0.33^a^	0.25^b^	0.31^a^	0.02	0.03	0.10	0.59
*PDK4*	0.34	0.31	0.78	0.37	0.35	0.03	0.98
Fatty acid oxidation, Lipoprotein and Cholesterol synthesis							
*ACOX1*	1.34	1.21	1.27	0.07	0.41	0.75	0.61
*APOB*	1.93	1.67	1.82	0.11	0.22	0.32	0.42
*BBOX1*	0.39	0.36	0.38	0.02	0.61	0.04	0.96
*CPT1A*	0.13	0.13	0.14	0.01	0.89	<.0001	0.49
*HMGCS2*	1.13	1.00	0.93	0.10	0.32	0.22	0.56
*MTTP*	1.48^a^	1.50^a^	1.28^b^	0.08	0.05	0.02	0.84
*PPARA*	0.43	0.40	0.46	0.02	0.16	<.0001	0.03
*SLC22A5*	2.89	2.92	3.08	0.29	0.88	<.0001	0.14
*TMLHE*	0.42	0.40	0.41	0.02	0.68	0.003	0.39
Hepatokines							
*ANGPTL4*	0.01	0.01	0.02	0.002	0.34	<.0001	0.03
*FGF21*	0.27	0.13	0.22	0.07	0.15	0.005	0.0006
Methionine cycle and methylation							
*BHMT*	1.13	0.96	1.02	0.11	0.54	0.002	0.70
*BHMT2*	0.38	0.45	0.37	0.04	0.20	0.21	0.57
*DNMT1*	0.02	0.02	0.02	0.001	0.53	0.03	0.64
*DNMT3A*	0.99^a^	1.26^b^	1.20^b^	0.09	0.04	0.05	0.91
*MAT1A*	1.46	1.55	1.52	0.08	0.71	0.09	0.86
*MTR*	0.05^a^	0.03^b^	0.04^ab^	0.002	0.01	0.31	0.65
*PEMT*	0.33	0.29	0.30	0.02	0.31	0.07	0.95
*SAHH*	1.39	1.25	1.39	0.06	0.17	0.0003	0.0009
Antioxidant system							
*CBS*	1.41	1.66	1.51	0.11	0.27	0.08	0.93
*CSAD*	0.23	0.26	0.22	0.04	0.64	0.0004	0.36
*CTH*	0.45	0.46	0.45	0.02	0.93	0.24	0.86
*GCLC*	0.06	0.06	0.05	0.004	0.80	0.01	0.35
*GPX1*	1.39	1.37	1.39	0.10	0.99	0.04	0.77
*GSR*	0.28	0.30	0.26	0.02	0.11	<.0001	0.11
GSS	0.45	0.49	0.46	0.02	0.34	0.78	0.95
*SOD1*	3.56	3.64	3.40	0.12	0.27	0.07	0.52
*SOD2*	4.06	4.08	4.10	0.21	0.99	0.92	0.53
Inflammatory response							
*CP*	2.48	2.13	2.37	0.22	0.49	0.003	0.85
*HP*	0.18	0.13	0.14	0.09	0.89	0.01	0.48
*NFKB1*	2.29	2.14	2.48	0.14	0.25	0.04	0.83
*RXRA*	0.57	0.56	0.58	0.03	0.86	0.20	0.66
*SAA2*	0.01	0.01	0.02	0.002	0.68	0.02	0.95
*SOCS2*	2.09	1.84	2.26	0.24	0.44	0.16	0.60
*STAT3*	1.38	1.42	1.39	0.11	0.96	0.05	0.43
*STAT5B*	2.30	2.26	2.45	0.07	0.15	0.002	0.07

^1^Prepartum dietary treatment: CON = control energy, OVE = moderate energy, OVE + SM = OVE supplemented with rumen-protected methionine (Smartamine M, Adisseo Inc.)

^2^SE = greatest standard error of the mean

^3^D = diet, T = time, D*T = diet by time interaction

^a, b^Significant difference among dietary groups (*P* ≤ 0.05). Differences reported for genes with a significant (*P* ≤ 0.05) Diet effect

## Results

### Blood biomarkers

#### Metabolism

**Fig. 2:**
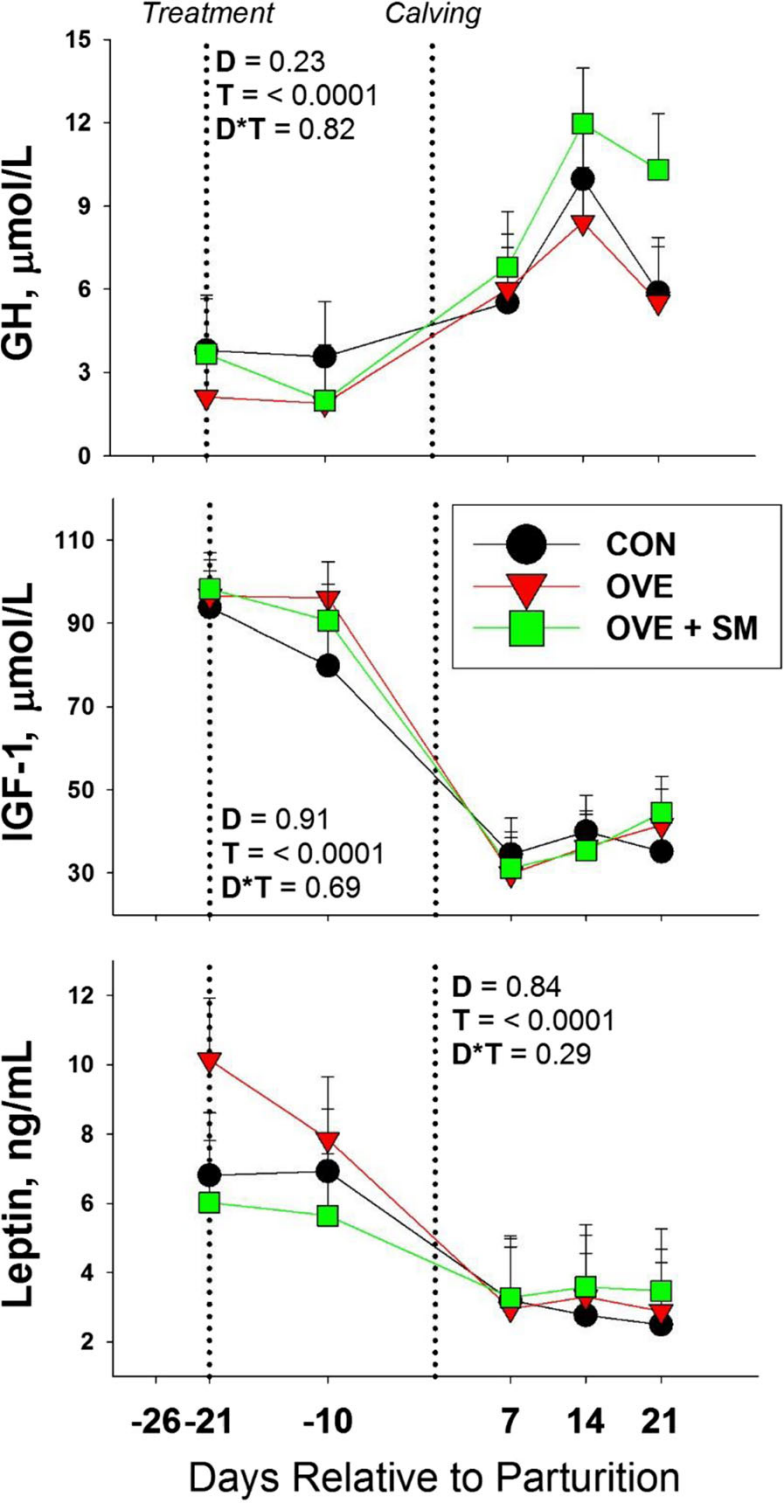
Effect of feeding a control lower-energy diet (CON, 1.24 Mcal/kg DM; high-straw) during the whole dry period (~50 d), a higher-energy (1.54 Mcal/kg DM) diet without (OVE) rumen-protected methionine during the last 21 d before calving, or OVE plus rumen-protected methionine (Smartamine M; OVE + SM; Adisseo NA) from -21 d before calving through the first 30 d postpartum on endocrine profiles in Holstein cows

Time affected all metabolic biomarkers (cholesterol, creatinine, GH, IGF1, leptin, urea; T, *P* < 0.001). However, no effect of diet or its interaction with time was detected (D, D*T, *P* > 0.05) (Fig. 2).

#### Health status

No effects of diet or its interaction with time were significant for haptoglobin or IL-6 concentration (D, D*T, *P* > 0.05). Diet affected albumin concentration (D*T, *P* < 0.05), with greater (*P* < 0.05) postpartum concentrations in OVE + SM compared with both other groups. The overall concentration of total bilirubin, ceruloplasmin, and serum amyloid A tended to be affected by diet (D, *P* < 0.10), with greater levels (*P* < 0.05) in OVE cows compared with CON (total bilirubin), OVE + SM (ceruloplasmin), or both other groups (SAA). Diet also affected GGT and GOT concentration, as OVE + SM had greater (*P* < 0.05) overall GGT concentration (D, *P* < 0.10), especially postpartum (d 14 and 21), compared with OVE, and lower (*P* < 0.05) GOT concentration postpartum (d 7 and 14) (D*T, *P* < 0.05) compared with CON cows. Time affected the concentration of all previous health biomarkers (T, *P* < 0.01).

#### Antioxidant and oxidative status

No effect of diet was detected for ORAC, total ROM and tocopherol (D, *P* > 0.05). Total NO_x_ also were not affected by diet, despite the fact that concentrations of both NO_2_ and NO_3_ had significant interactions or diet effects (NO_2_, D*T, *P* < 0.10; NO_3_, D, *P* < 0.05, D*T, *P* < 0.10). Diet had a strong effect on GSH concentration (D, *P* < 0.001), with greatest concentration (*P* < 0.05) in OVE + SM cows compared with both other groups. When interacting with time, diet tended to affect blood concentration of β-carotene and retinol (D*T, *P* < 0.10). For the first, the response was due to a greater (*P* < 0.05) concentration in OVE + SM cows compared with CON at -21 and -10 d, and to a lower (*P* < 0.05) concentration in OVE compared with CON at 14 d relative to parturition. In the case of retinol, the interaction was due the increasing (*P* < 0.05) concentration postpartum from 7 to 21 d in OVE + SM cows, while in CON and OVE cows the concentration remained constant (*P* > 0.05). This led to a greater (*P* < 0.05) retinol concentration in OVE + SM at 21 d postpartum compare with OVE. Diet also affected paraoxonase concentration, with overall greater level (*P* < 0.05) in CON compared with OVE and OVE + SM (D, *P* < 0.05). This difference was due to greater (*P* < 0.05) concentration in CON cows at -21, -10 and 7 d relative to parturition (D*T, *P* < 0.05).

### Gene expression

#### Energy metabolism

Cows fed the CON or OVE + SM diets had greater *PCK1* expression compared with OVE cows (D, *P* < 0.05). Diet also affected the expression of the fatty acid metabolism related genes *MTTP* (D, *P* < 0.05) and *PPARA* (D*T, *P* < 0.05). Expression of *MTTP* was in fact greater (*P* < 0.05) in CON and OVE cows, compared with OVE + SM, while *PPARA* expression was greater (*P* < 0.05) prepartum (-10 d) for OVE + SM compared with CON and OVE, and lower (*P* < 0.05) early postpartum (7 d) for OVE compared with the other two groups.

#### Hepatokines and inflammation

Diet alone did not affect genes related to hepatokiens and the inflammatory response (D, *P* > 0.05). However, the hepatokines *ANGPTL4* and *FGF21* had a significant interaction with time (D*T, *P* < 0.05). For *FGF21* this significance was due to a greater (*P* < 0.05) prepartal expression in CON and OVE + SM compared with OVE cows, while for *ANPTL4* no differences among dietary groups were detected across the analyzed time points (*P* > 0.05).

#### Methionine cycle and antioxidant system

No genes concerning the antioxidant system were significantly affected by diet, or its interaction with time (D, D*T, *P* > 0.05). However, *MTR* and *DNMT3A*, genes of the methionine cycle, had an overall effect of diet (D, *P* < 0.05). Expression of *MTR* was greater (*P* < 0.05) in CON compared with OVE, with OVE + SM having an intermediate level of expression, while *DNMT3A* expression was greater (*P* < 0.05) in OVE and OVE + SA compared with CON cows. Furthermore, *SAHH* expression was greater (D*T, *P* < 0.05) prepartum in OVE + SM cows compared with the other dietary groups; whereas, expression was greater (*P* < 0.05) early postpartum (7 d) in CON cows compared with OVE and OVE + SM.

## Discussion

Overfeeding dairy cows in the weeks prior parturition (e.g. close up period) has been previously linked with a more pronounced negative energy balance postpartum, due to bigger drops in voluntary dry matter intake (DMI) along with sustained lipid mobilization and possible accumulation of triacylglycerol (TAG) in the liver [25]. The present study confirmed the overfeeding-induced depression of DMI postpartum and hepatic TAG accumulation [25]. Furthermore, despite previous studies reporting that overfed cows were always able to maintain similar levels of milk production as the control-fed counterparts [31], these changes led to worse milk performance including lower milk and energy corrected milk yield [25].

As hypothesized, supplementation of rumen-protected Met to a moderate energy diet was able to overcome the detrimental effects of energy overfeeding. In fact, OVE + SM cows compared with OVE had greater postpartal DMI and better milk production, matching the performance of the control-fed group [25]. Despite the fact that the improved DMI, likely a consequence of the improved health status, could easily explain the improved production performance, other cellular and physiologic also likely were contributing factors.

The hepatic transcriptome revealed how Met supplementation restored *PCK1* expression (an important gluconeogenic gene) to the level of control-fed cows. At least postpartum this could be explained by the higher insulin concentration in OVE + SM [25], as hepatic *PCK1* mRNA expression is directly related to insulin level [32]. The increased insulin concentration also could explain why circulating glucose was lower in OVE + SM cows [25] compared with CON, i.e. overfeeding alone does not affect peripheral insulin resistance [9], and the increased insulin concentration was not followed by changes in GH or IGF1, hence, the improved milk production with OVE + SM also might have resulted from an increase in glucose availability directly channeled to peripheral tissues and the mammary gland. In the latter case it would have contributed to greater lactose production. Peripheral tissues, i.e. adipose and muscle, rely mainly on GLUT4 (an insulin-dependent transporter) for glucose uptake, while the mammary gland uses mainly GLUT1 (usually described as insulin-independent) as the preferred glucose transporter [33]. However, a recent study revealed that insulin increases GLUT1 expression in bovine mammary explants, thus, providing evidence of a functional link between circulating insulin and mammary glucose uptake [34].

Supplementing Met also increased both fat and protein percentage during the first week of lactation [25]. Because biomarkers of muscle catabolism were not affected by diet (e.g. urea and creatinine) and DMI was similar in CON and OVE + SM, we speculate that Met itself, combined with higher circulating insulin, might have been the primary cause of the improved protein percentage. In fact, previous research demonstrated that an increase in amino acid supply (e.g. abomasal casein infusion) could markedly improve milk protein yield, especially when the circulating level of insulin was artificially raised through a clamp [35, 36]. The lower inflammation status and greater liver function around calving in the OVE + SM cows (lower concentrations of albumin and greater bilirubin, ceruloplasmin, GGT, GOT, and SAA) would have guaranteed higher availability of plasma amino acids [37] to the mammary gland for protein synthesis. The increase in fat content, which agrees with several previous studies [16, 38–41], might have been related to cellular pathways involving Met and its methylated compounds (e.g. choline [42]), which some data indicate are important for supporting milk fat synthesis in cows [43].

**Fig. 3:**
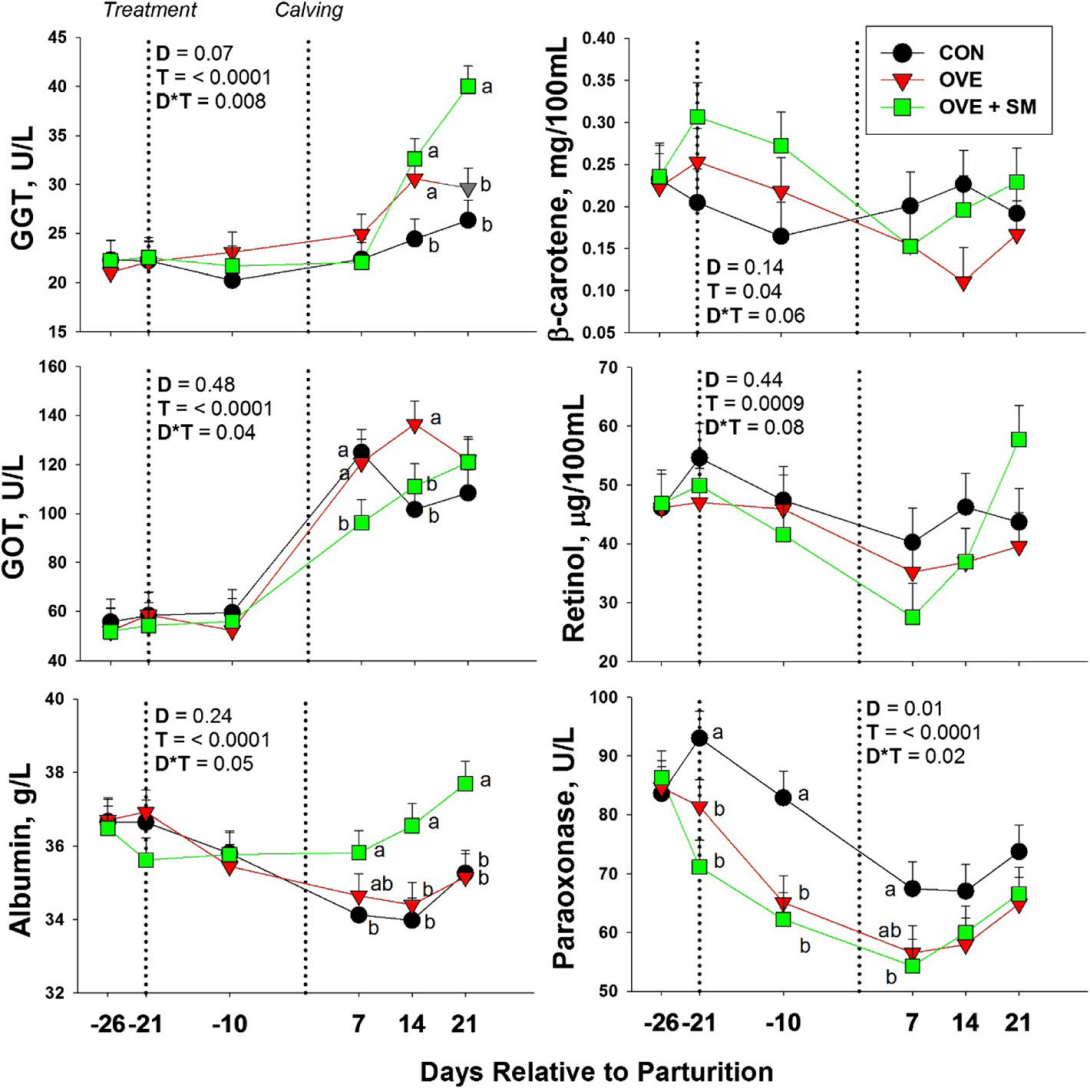
Effect of feeding a control lower-energy diet (CON, 1.24 Mcal/kg DM; high-straw) during the whole dry period (~50 d), a higher-energy (1.54 Mcal/kg DM) diet without (OVE) rumen-protected methionine during the last 21 d before calving, or OVE plus rumen-protected methionine (Smartamine M; OVE + SM; Adisseo NA) from -21 d before calving through the first 30 d postpartum on blood biomarkers of liver function and antioxidant status in Holstein cows

As previously mentioned, overfeeding energy prepartum led to hepatic TAG accumulation [25], a condition that, if excessive, could become a potential burden for proper liver function [2]. OVE cows, in fact, had signs of impaired liver function and inflammatory condition postpartum including lower concentrations of albumin and greater bilirubin, ceruloplasmin, GGT, GOT, and SAA (Table 2, Fig. 3). As hypothesized, supplemental Met was able to correct these effects of the OVE diet. Thus, as a primary outcome, OVE + SM cows had less liver TAG accumulation [25] despite similar NEFA concentration between OVE and OVE + SM [25]. This was at least in part due to greater *PPARA* expression with Met supplementation.

**Table 2:** Effect of feeding a control lower-energy diet (CON, 1.24 Mcal/kg DM; high-straw) during the whole dry period (~50 d), a higher-energy (1.54 Mcal/kg DM) diet without (OVE) rumen-protected methionine during the last 21 d before calving, or OVE plus rumen-protected methionine (Smartamine M; OVE + SM; Adisseo NA) from -21 d before calving through the first 30 d postpartum on biomarker concentrations of metabolism, liver health, and oxidative status in Holstein cows

	Diet^1^				*P*-values^3^		
Items	CON	OVE	OVE + SM	SE^2^	D	T	D*T
Metabolism							
Cholesterol, mmol/L	3.24	3.16	3.26	0.11	0.76	<.0001	0.71
Creatinine, μmol/L	97.60	98.88	97.68	1.53	0.77	<.0001	0.17
GH, ng/mL	5.75	4.79	6.95	1.08	0.23	<.0001	0.82
IGF-1, ng/mL	56.65	60.03	59.98	6.64	0.91	<.0001	0.69
Leptin, ng/mL	4.44	5.42	4.40	1.62	0.84	<.0001	0.29
Urea, mmol/L	5.20	5.05	5.05	0.18	0.77	<.0001	0.30
Liver health							
Albumin, g/L	35.41	35.54	36.32	0.41	0.24	0.0002	0.05
Bilirubin , μmol/L	2.29^a^	3.38^b^	2.57^ab^	0.41	0.10	<.0001	0.57
Ceruloplasmin, μmol/L	2.77^ab^	2.91^b^	2.61^a^	0.09	0.006	<.0001	0.51
GGT, U/L	22.96^a^	25.21^ab^	26.85^b^	1.17	0.07	<.0001	0.008
GOT, U/L	84.76	90.30	81.71	5.61	0.48	<.0001	0.04
Haptoglobin, g/L	0.42	0.46	0.41	0.06	0.76	0.003	0.86
IL-6, pg/mL	530.63	586.37	412.76	98.56	0.37	0.001	0.67
SAA, μg/mL	35.55^a^	54.00^b^	34.77^a^	7.79	0.10	0.0005	0.58
Oxidative status							
β-carotene, mg/100 mL	0.20	0.19	0.23	0.02	0.14	0.04	0.06
Liver GSH, mmol/L	953^a^	1281^b^	1693^c^	120	0.0002	0.05	0.14
NO_2_, μmol/L	6.03	6.66	6.80	0.45	0.44	0.01	0.09
NO_3_, μmol/L	18.65^a^	16.90^b^	16.77^b^	0.40	0.002	<.0001	0.08
NO_x_, μmol/L	24.61	23.54	23.67	0.56	0.31	<.0001	0.18
ORAC, TE mol/L	12,731	12,359	12,739	198	0.25	<.0001	0.66
Paraoxonase, U/mL	77.96^a^	68.41^b^	66.74^b^	2.68	0.01	<.0001	0.02
Retinol, μg/100 mL	46.39	41.79	43.42	3.10	0.44	0.0009	0.08
ROM, mg of H_2_O_2_/100 mL	14.01	12.99	13.44	0.49	0.31	<.0001	0.79
Tocopherol, μg/mL	3.67	3.68	3.16	0.44	0.46	<.0001	0.31

^1^Prepartum dietary treatment: CON = control energy, OVE = moderate energy, OVE + SM = OVE supplemented with rumen-protected methionine (Smartamine M, Adisseo Inc.)

^2^SE = greatest standard error of the mean

^3^D = diet, T = time, D*T = diet by time interaction

^a, b, c^Significant difference among dietary groups (*P* ≤ 0.05). Differen reported for biomarkers with a tendency (*P* ≤ 0.10) or a significan (*P* ≤ 0.05) Diet effect

Among the most important metabolic functions coordinated by PPARα are LCFA uptake, intracellular activation, oxidation, and ketogenesis [44]. Thus its greater expression in OVE + SM cows could have improved NEFA handling, i.e. through greater oxidation. Furthermore, PCK1 is also involved in glyceroneogenesis, as it can catalyze the production of glycerol-3-phospate for use during fatty acid esterification [45]. Thus the increase of its expression could have further improved NEFA handling by the liver. The lower expression of *MTTP* in the OVE + SM cows was lower indicated a potentially lower capacity of these cows to synthesize and export VLDL. However, the data from Bernabucci et al. [46] indicated that apolipoprotein mRNA transcription rather than *MTTP* might be the limiting step in the repackaging of TAG into lipoproteins, hence, explaining the increase in concentration of plasma VLDL in OVE + SM cows [25]. As a subsequent outcome, the improved fatty acid metabolism in liver with Met supplementation reduces the risk of liver dysfunction, an idea supported by the biomarkers of liver function (e.g. greater albumin and VLDL, and lower bilirubin) in OVE + SM cows [47].

Metabolic dysfunction and inflammatory events are often linked through oxidative stress, a common outcome to both scenarios [48–50]. The present study partly confirmed the possible molecular mechanisms through which prepartum overfeeding could cause an increased concentration of oxidants proposed by Loor et al. [51]. OVE did not cause changes in total ROM and NO_x_, however, these cows had an impairment of the antioxidant system. Despite similar blood antioxidant capacity, paraoxonase concentration was in fact lower in OVE cows, a condition that not only indicates liver dysfunction, but one that has been proven to lead to an increase in the inflammatory status (confirmed by higher ceruloplasmin and SAA), which notoriously causes an increase in oxidative stress, and a reduction of antioxidative protection during the early postpartum period [27, 52]. As for paraoxonase, postpartum (d 14) concentration of β-carotene, a precursor of vitamin A, which exerts antioxidant effects [53], also was reduced in OVE compared with CON.

Supplementation of rumen-protected Met has been proven to benefit the oxidative status of periparturient cows [19, 20], in large part because it is a precursor for the biosynthesis of glutathione and taurine, two of the most important cellular antioxidants [54, 55]. In the present study, Met supplementation to cows fed a higher energy diet prepartum was able to improve their compromised antioxidant status. In fact, despite the lack of changes in ROM or paraoxonase compared with OVE, OVE + SM cows had greater glutathione concentrations, even compared with CON, together with higher retinol concentrations up to the level of control-fed cows. Concerning retinol, its concentration is also regulated by the hepatic synthesis of its carrier, retinol binding protein [56]. Thus, a greater plasma retinol concentration, besides suggesting a better antioxidant status, could also be a response to the better liver functionality detected in OVE + SM cows. Furthermore, *GSR* expression was decreased in OVE + SM cows to a similar level than OVE. *GSR* encodes the protein glutathione reductase, a central enzyme of cellular antioxidant defense, which reduces oxidized glutathione disulfide to the sulfhydryl form [57]. This further suggests a lesser oxidative status in cows fed methionine, which despite having a greater glutathione concentration seemed to have less of a need to restore the pool of its active form.

Other health benefits of methionine supplementation could also be noticed in the lower somatic cell count in milk. For instance, OVE + SM cows compared with both CON and OVE had lower milk SCC [25], a result that further highlights the immunometabolic effects of methionine and its metabolites [16, 21–23].

At a molecular level, the greater expression *SAHH* prepartum in OVE + SM cows underscores that the increased Met supply to the liver through supplementation was directed through the methionine cycle, leading to the higher glutathione concentrations. However, overfeeding energy prepartum (e.g. OVE and OVE + SM) seemed to reduce the overall expression of *MTR*, as if regenerating Met was not a hepatic priority. This becomes relevant in early lactation, because after calving the decrease in expression of both *MTR* and *SAHH* in all groups indicated that cows might redirect the circulating Met to the mammary gland for milk production. To further complicate this scenario, the greater *DNMT3A* expression in both OVE and OVE + SM cows indicated a role of overfeeding in its regulation. Its greater expression could indicate a higher need of methyl groups from methionine by the liver, hence, in light of the lower hepatic regeneration (e.g. lower *MTR*) but greater utilization (e.g. higher *DNMT3A*) Met supplementation (e.g. OVE + SM) favored the mammary demand. The fact that milk production was restored to the level of CON cows in the OVE + SM cows supports this scenario.

The mechanisms by which prepartal overfeeding causes a greater *DNMT3A* expression, increasing DNA methylation and leading to greater consumption of methyl groups from Met, are not clear. Insulin sensitivity was previously associated with increased global methylation [58], but overfeeding cows prepartum never led to its impairment in our previous experiments [9, 10]. On the other hand, levels of hepatic methylation were associated with fatty liver disease in humans [59, 60]. Because OVE cows had a greater hepatic TAG content [25], *DNMT3A* expression regulation could be explained by the alterations in lipid metabolism.

## Conclusions

Current results confirm the detrimental outcome (e.g., reduced DMI, compromised liver function, and higher inflammatory status) of a higher-energy diet during the close up period in dairy cows, thus, supporting the need for energy restriction in the close-up period. However, if the practice persists, dairy producers should improve the diet methionine supply. In fact, supplemental rumen-protected methionine was effective in reducing the aforementioned effects, by (i) stimulating DMI and milk production, (ii) improving hepatic fatty acid metabolism and reducing TAG accumulation, (iii) improving general biomarkers of liver function, and (iv) limiting the postpartal negative effect of inflammation on the cow antioxidant system. Further investigation is needed to assess the effect of methionine supplementation to a prepartal energy restricted diet during the close-up.

## Additional file

Additional file 1: Complete gene expression methodology, including primer sequences and qPCR performance. Figures S1-S5 include the two-way interaction graphs not shown in the manuscript. (DOCX 8109 kb)

## Abbreviations

ACOX1: Acyl-CoA oxidase 1
ANGPTL4: Angiopoietin like 4
APOB: Apolipoprotein B
BBOX1: γ-butyrobetaine hydroxylase 1
BHMT: BHMT2, Betaine--homocysteine S-methyltransferase 1, and 2
CBS: Cystathionine-beta-synthase
CP: Ceruloplasmin
CPT1A: Carnitine palmitoyltransferase 1A
CSAD: Cysteine sulfinic acid decarboxylase
CTH: Cystathionine gamma-lyase
DMI: Dry matter intake
DNMT1: DNMT3A, DNA (cytosine-5-)-methyltransferase 1, and 3 α
FGF21: Fibroblast growth factor 21
GCLC: Glutamate-cysteine ligase catalytic subunit
GGT: Gamma-glutamyl-transpeptidase
GH: Growth hormone
GLUT1: GLUT4, Glucose transporter 1, and 4
GOT: Glutamic oxaloacetic transaminase
GPX1: Glutathione peroxidase 1
GSR: Glutathione reductase
GSS: Glutathione synthetase
HMGCS2: 3-hydroxy-3-methylglutaryl-CoA synthase 2
HP: Haptoglobin
IGF1: Insulin like growth factor-1
IL6: Interleukin 6
MAT1A: Methionine adenosyltransferase 1A
Met: Methionine
MTR: 5-methyltetrahydrofolate-homocysteine methyltransferase
MTTP: Microsomal triglyceride transfer protein
NEFA: Non-esterified fatty acids
NEL: Net energy for lactation
NFKB1: Nuclear factor κB subunit 1
NOx: NO2, NO3, Nitric oxides
ORAC: Oxygen radical absorbance capacity
PC: Pyruvate carboxylase
PCK1: Phosphoenolpyruvate carboxykinase 1
PDK4: Pyruvate dehydrogenase kinase 4
PEMT: Phosphatidylethanolamine N-methyltransferase
PPARA: Peroxisome proliferator activated receptor α
ROM: Reactive oxygen metabolites
RXRA: Retinoid X receptor α
SAA: SAA2, Serum amyloid A, and A2
SAHH: S-adenosylhomocysteine hydrolase
SLC22A5: Solute carrier family 22 member 5
SOCS2: Suppressor of cytokine signaling 2
SOD1: SOD2, Superoxide dismutase 1 (soluble), and 2 (mitochondrial)
STAT3: STAT5B, Signal transducer and activator of transcription 3, and 5B
TAG: Triacylglycerol
TMLHE: Trimethyllysine hydroxylase, ε
VLDL: Very low density lipoprotein
