# Supplementary material for: Supplemental Smartamine M in higher-energy diets during the prepartal period improves hepatic biomarkers of health and oxidative status in Holstein cows
Source: J Anim Sci Biotechnol. 2017 Feb 6;8:17. doi: 10.1186/s40104-017-0147-7 (PMC5295218; doi:10.1186/s40104-017-0147-7)
Supplement: Additional file 1: — Complete gene expression methodology, including primer sequences and qPCR performance. Figures S1-S5 include the two-way interaction graphs not shown in the manuscript. (DOCX 8109 kb) [file 40104_2017_147_MOESM1_ESM.docx]

**ADDITIONAL FILE 1**

**Supplemental Smartamine M in higher-energy diets during the prepartal period improves hepatic biomarkers of health and oxidative status in Holstein cows.**

Vailati-Riboni et al., 2016

***RNA Extraction.*** Adipose tissue was weighted (~0.2 g), immediately placed in 1.2 mL of QIAzol Lysis Reagent (cat#79306, Qiagen) and homogenized using a Mini-Beadbeater-24 (cat#112011, Biospec Products Inc.) with two 30 s cycles, and 1 min incubation on ice in between the cycles. Samples were then centrifuged for 10 min at 12,000 g and 4 °C, and the supernatant was transfer in a separate tube and mix with 240 μL of Chloroform (cat#C298, Fisher Chemical). After centrifugation for 15 min at 12,000 g and 4 °C, the aqueous phase was transferred to a new tube, mixed with 900 μL of 100% Ethanol (cat#2701, Decon Labs), and total RNA was cleaned using miRNeasy mini kit columns (cat# 217004, Qiagen) following manufacture protocol. During purification, genomic DNA was removed using the RNase-Free DNase Set (cat# 79254, Qiagen).

***RNA Quality Evaluation.*** The RNA quantity, purity, and integrity were determined using a NanoDrop ND-1000 (cat#E112352, NanoDrop Technologies Inc.) and the Agilent 2100 Bioanalyzer (cat#G2939AA, Agilent Technologies Inc.). Samples had an average RIN score of 6.5 ± 0.8. RNA samples were stored at -80 °C until analysis.

***mRNA cDNA Synthesis and qPCR Performance.*** Complete information about primer sequences and qPCR performance are reported in Supplemental Table 1and 2. A portion of the RNA was diluted to 100 ng/μL with DNase/RNase-free water for cDNA synthesis through RT-PCR. Per each sample, 4 μL of diluted RNA, 5 μL of Random Primers (3 μg/μL; cat#11034731001, Roche) and 45 μL of DNase/RNase-free water. The mixture was incubated at 65°C for 5 min and kept on ice for 3 min. A total of 36 μL of master mix, composed of 20 μL of 5X First-Strand Buffer (cat#EP0441, Thermo Scientific), 5 μL of Oligo dT18 (Custom DNA Oligo Tubes, Integrated DNA Technologies), 10 μL of 10mM dNTP mix (cat#18427-088, Invitrogen), 1.25 μL of Reverse Transcriptase (200 U/μL as final concentration, cat#EP0441, Thermo Scientific), 0.625 μL of Rnase inhibitor (200 U/μL as final concentration, cat#EO0381, Thermo Scientific), and 8.125 μL of DNase/RNase-free water, was added. The reaction was performed in an Eppendorf Mastercycler Gradient following the appropriate temperature program: 25°C for 5 min, 42°C for 60 min, and 70°C for 5 min. The cDNA was then diluted 1:4 with DNase/RNase-free water, prior to quantitative PCR analysis.

Quantitative PCR was performed using 4 μL diluted cDNA combined with 6 μL of a mixture composed of 5 μL SYBR Green master mix (cat#95073-05K, Quanta BioSciences), 0.4 μL each of 10 μM forward and reverse primers, and 0.2 μL DNase/RNase free water in a MicroAmp™ Optical 384-Well Reaction Plate (Applied Biosystems). Each sample was run in triplicate and a 7 point relative standard curve plus the non-template control were used. The reactions were performed in an ABI Prism 7900 HT SDS instrument using the following conditions: 2 min at 50 °C, 10 min at 95 °C, 40 cycles of 15 s at 95 °C (denaturation), and 1 min at 60 °C (annealing + extension). The presence of a single PCR product was verified by the dissociation protocol using incremental temperatures to 95 °C for 15 s plus 65 °C for 15 s. Data were calculated with the 7900 HT Sequence Detection Systems Software. The final data were normalized using the geometric mean of three internal control genes (ICG): *GAPDH*, *RPS9,* and *UXT* [1, 2].

**Table S1.** Gene symbol, accession number, and forward and reverse primer sequences of genes analyzed in liver.

| **Gene** | **Accession #** | **Forward Primer** | | **Reverse Primer** |
| --- | --- | --- | --- | --- |
| *ACOX1* | NM_001035289.2 | ACCCAGACTTCCAGCATGAGA | | TTCCTCATCTTCTGCACCATGA |
| *ANGPTL4* | NM_001046043.2 | AGGAAGAGGCTGCCCAAGAT | | CCCTCTCTCCCTCTTCAAACA |
| *APOB* | XM_002691511.2 | CAAGGCTTTGTACTGGGTTAAGG | | ACCATGTCCTGCTCATGTTTATCA |
| *BBOX1* | BC102355.1 | TCCAGCTGCCTACTCTGGAT | | AGCTGAACCTTACCCCAGGT |
| *BHMT* | NM_001011679.1 | GCTCTCCTCGTCCATCCTCAT | CCGTTCTAGGATGCCCTTCTT | |
| *BHMT2* | XM_003586514.2 | TGCAACCAGATGGGATATTCAA | TCTGATGTGATAGGGCTCAAAGC | |
| *CBS* | NM_001102000.2 | GCCACCACCTCTGTCAAATTC | GGACAGAAAGCAGAGTGGTAACTG | |
| *CP* | NM_001256556.1 | GGTTGACACGGAACATTCCAA | GGCCTAAAAACCCTAACCAGACA | |
| *CPT1A* | FJ415874.1 | TCGCGATGGACTTGCTGTATA | CGGTCCAGTTTGCGTCTGTA | |
| *CSAD* | XM_005206242.1 | GACACCTCGAACCTGCTCAAG | CTGTGTCCAGAGCCACATCGT | |
| *CTH* | NM_001024567.1 | AAGTCCGCATGGAGAAGCATT | GAAGGCAGCCCAGGATAAATAA | |
| *DNMT1* | NM_182651.2 | GGTGAGGATTGCAGAGTGGTAAT | CTTCAGACTTTGTTTCTCCATCAGACT | |
| *DNMT3A* | NM_001206502.1 | AGCGAACCTCATCATTGGAATAA | GCTGTGAGCTTACTCCTGAGCAA | |
| *FGF21* | XM_002695200.2 | CAGAGCCCCGAAAGTCTCTTG | AAAGTGCAGCGATCCGTACAG | |
| *GCLC* | NM_001083674.1 | GGCGTCCGGAGAATTAATGAC | TCAGTTATTACACTGTCCTGCTTGTAGTC | |
| *GPX1* | NM_174076.3 | AACGCCAAGAACGAGGAGATC | CATTCACCTCGCACTTTTCGA | |
| *GSR* | NM_001114190.2 | CGCTGAGAACCCAGAGACTTG | AAACGGAAAGTGGGAACAGTAAGTA | |
| *GSS* | NM_001015630.1 | CGAGTGATCCAATGCATTTCAG | ATGTCCCACGTGCTTGTTCAT | |
| *HMGCS2* | NM_001045883.1 | TTACGGGCCCTGGACAAAT | GCACATCATCGAGAGTGAAAGG | |
| *HP* | NM_001040470.1 | GGTTCGGAAAACCATCGCTA | CACTCGTGTCCCCTCCACTC | |
| *IGF1* | NM_001077828.1 | CCAATTCATTTCCAGACTTTGCA | CACCTGCTTCAAGAAATCACAAAA | |
| *MAT1A* | NM_001046497 | CAGATTCCAGATTCCAGT | CAAGGGCTTTGACTTTAAG | |
| *MTR* | NM_001030298.1 | GTTCCACCTGCCACTGTTTTC | CAGCGCTCTCCAATGTTAACAA | |
| *MTTP* | NM_001101834.1 | ACCAGGCTCATCAAGACAAAGTG | GTGACACCCAAGACCTGATGTG | |
| *NFKB1* | NM_001076409.1 | TTCAACCGGAGATGCCACTAC | ACACACGTAACGGAAACGAAATC | |
| *PC* | NM_177946 | CCTGCAGGGACCCAAGATT | TGGCCAAGGCTTTGATGTG | |
| *PCK1* | JQ733520.1 | AAGATTGGCATCGAGCTGACA | GTGGAGGCATTGACGAACTC | |
| *PDK4* | NM_001101883 | ATGTTCCATCTCACCTTCACCAT | AACTGTGGCCCTCATTGCAT | |
| *PEMT* | NM_182989.3 | TGTTCGTCCTTTCCAGTTTCCT | CTGAACGGGAACATGGTCACT | |
| *PPARA* | NM_001034036.1 | CATAACGCGATTCGTTTTGGA | CGCGGTTTCGGAATCTTCT | |
| *RXRA* | XM_881943.5 | TGTCCCCGATGAGCTTGAAG | GAGGCGTACTGCAAACACAAGT | |
| *SAA2* | NM_001075260.2 | GACCAGTGCTCTCCTCCACTG | CCCCTTCATAAGCCTCACCAA | |
| *SAHH* | NM_001034315.1 | TGTCAGGAGGGCAACATCTTT | AGTGCCCAATGTTACACACAATG | |
| *SLC22A5* | NM_001046502.2 | CACAGTGGTCAGGAACATGG | AATGGTGTCTGGGAGTGGAG | |
| *SOCS2* | NM_177523.2 | CGGCACTGTTCACCTTTATCTG | GACGGTGCTGGTACACTTGTTAAT | |
| *SOD1* | XM_005201085.1 | GGCTGTACCAGTGCAGGTCC | GCTGTCACATTGCCCAGGT | |
| *SOD2* | NM_201527.2 | TGTGGGAGCATGCTTATTACCTT | TGCAGTTACATTCTCCCAGTTGA | |
| *STAT3* | NM_001012671.2 | GGTAGCATGTGGGATGGTCTCT | GCATCCCTAGAAACTCTGGTCAA | |
| *STAT5B* | NM_174617.3 | TCATCAGATGCAAGCGCTGTA | TTATCAAGATCTATTGAGTCCCAAGCT | |
| *TMLHE* | NM_001076064.1 | TGGCAGGACACTGCTAGTTG | GACAGCCCGGTCATAGTTGT | |

**Table S2.** qPCR performance of measured hepatic genes.

| **Gene** | **Median Ct^1^** | **Median ∆Ct^2^** | **Slope^3^** | **(R^2^)^4^** | **Efficiency**^5^ | **relative mRNA abundance^6^** | **1/E∆Ct^7^** |
| --- | --- | --- | --- | --- | --- | --- | --- |
| *ACOX1* | 19.15 | -1.38 | -3.24 | 0.996 | 2.037 | 2.673 | 5.394 |
| *ANGPTL4* | 25.18 | 4.71 | -3.20 | 0.992 | 2.055 | 0.034 | 0.068 |
| *APOB* | 18.14 | -2.40 | -3.23 | 0.997 | 2.041 | 5.553 | 11.207 |
| *BBOX1* | 22.56 | 2.07 | -3.10 | 0.997 | 2.104 | 0.215 | 0.434 |
| *BHMT* | 18.96 | -1.50 | -3.36 | 0.992 | 1.984 | 2.791 | 5.634 |
| *BHMT2* | 24.11 | 3.56 | -2.93 | 0.993 | 2.195 | 0.061 | 0.123 |
| *CBS* | 21.59 | 1.13 | -3.26 | 0.993 | 2.028 | 0.451 | 0.911 |
| *CP* | 17.97 | -2.54 | -3.21 | 0.996 | 2.048 | 6.196 | 12.506 |
| *CPT1A* | 27.65 | 7.17 | -2.79 | 0.981 | 2.284 | 0.003 | 0.005 |
| *CSAD* | 24.47 | 3.92 | -3.27 | 0.996 | 2.024 | 0.063 | 0.127 |
| *CTH* | 25.45 | 5.04 | -3.12 | 0.996 | 2.094 | 0.024 | 0.049 |
| *DNMT1* | 27.68 | 7.07 | -2.97 | 0.988 | 2.174 | 0.004 | 0.008 |
| *DNMT3A* | 27.27 | 6.83 | -2.96 | 0.989 | 2.174 | 0.005 | 0.010 |
| *FGF21* | 25.78 | 5.30 | -3.24 | 0.992 | 2.037 | 0.023 | 0.046 |
| *GCLC* | 25.40 | 4.82 | -3.12 | 0.990 | 2.093 | 0.028 | 0.057 |
| *GPX1* | 20.91 | 0.41 | -3.06 | 0.998 | 2.125 | 0.732 | 1.477 |
| *GSR* | 25.28 | 4.79 | -3.12 | 0.995 | 2.091 | 0.029 | 0.059 |
| *GSS* | 25.98 | 5.47 | -3.12 | 0.996 | 2.094 | 0.018 | 0.035 |
| *HMGCS2* | 18.32 | -2.22 | -3.18 | 0.998 | 2.062 | 4.996 | 10.084 |
| *HP* | 20.97 | 0.45 | -3.26 | 0.997 | 2.025 | 0.727 | 1.468 |
| *IGF1* | 22.88 | 2.42 | -3.33 | 0.995 | 1.996 | 0.188 | 0.380 |
| *MAT1A* | 19.47 | -1.02 | -3.25 | 0.995 | 2.030 | 2.053 | 4.143 |
| *MTR* | 26.37 | 5.92 | -3.30 | 0.981 | 2.008 | 0.016 | 0.033 |
| *MTTP* | 22.22 | 1.66 | -3.12 | 0.993 | 2.092 | 0.293 | 0.591 |
| *NFKB1* | 23.83 | 3.38 | -3.10 | 0.996 | 2.102 | 0.081 | 0.164 |
| *PC* | 23.08 | 2.71 | -3.59 | 0.996 | 1.899 | 0.176 | 0.355 |
| *PCK1* | 19.88 | -0.66 | -3.22 | 0.996 | 2.046 | 1.609 | 3.247 |
| *PDK4* | 23.39 | 2.92 | -3.09 | 0.996 | 2.107 | 0.113 | 0.229 |
| *PEMT* | 22.78 | 2.28 | -3.23 | 0.991 | 2.039 | 0.197 | 0.397 |
| *PPARA* | 22.41 | 1.96 | -3.21 | 0.999 | 2.047 | 0.245 | 0.494 |
| *RXRA* | 22.86 | 2.34 | -3.36 | 0.991 | 1.983 | 0.201 | 0.406 |
| *SAA2* | 19.13 | -1.41 | -3.27 | 0.994 | 2.020 | 2.689 | 5.428 |
| *SAHH* | 17.37 | -3.07 | -3.24 | 0.998 | 2.037 | 8.859 | 17.879 |
| *SLC22A5* | 25.86 | 5.29 | -3.08 | 0.994 | 2.111 | 0.019 | 0.039 |
| *SOCS2* | 23.40 | 2.85 | -3.00 | 0.994 | 2.153 | 0.112 | 0.226 |
| *SOD1* | 18.15 | -2.40 | -3.13 | 0.997 | 2.088 | 5.840 | 11.787 |
| *SOD2* | 19.88 | -0.60 | -3.13 | 0.994 | 2.088 | 1.553 | 3.135 |
| *STAT3* | 21.52 | 1.04 | -3.35 | 0.981 | 1.990 | 0.489 | 0.988 |
| *STAT5B* | 23.07 | 2.55 | -3.14 | 0.988 | 2.083 | 0.154 | 0.310 |
| *TMLHE* | 25.46 | 4.93 | -3.29 | 0.991 | 2.014 | 0.032 | 0.064 |
| ^1^ The median is calculated considering all time points and all cows.  ^2^ The median of ∆Ct is calculated as [Ct gene – geometrical mean of Ct internal controls] for each time point and each cow.  ^3^ Slope of the standard curve.  ^4^ R^2^ stands for the coefficient of determination of the standard curve.  ^5^ Efficiency is calculated as [10^(-1 / Slope)^].  ^6^ relative mRNA abundance = 1/ Efficiency ^Median ∆Ct^  ^7^1/E∆Ct = relative mRNA abundance/∑relative mRNA abundance | | | | | | | |

**Figure S1.** Effect of prepartum diet on metabolic biomarker in dairy cows during the transition period.


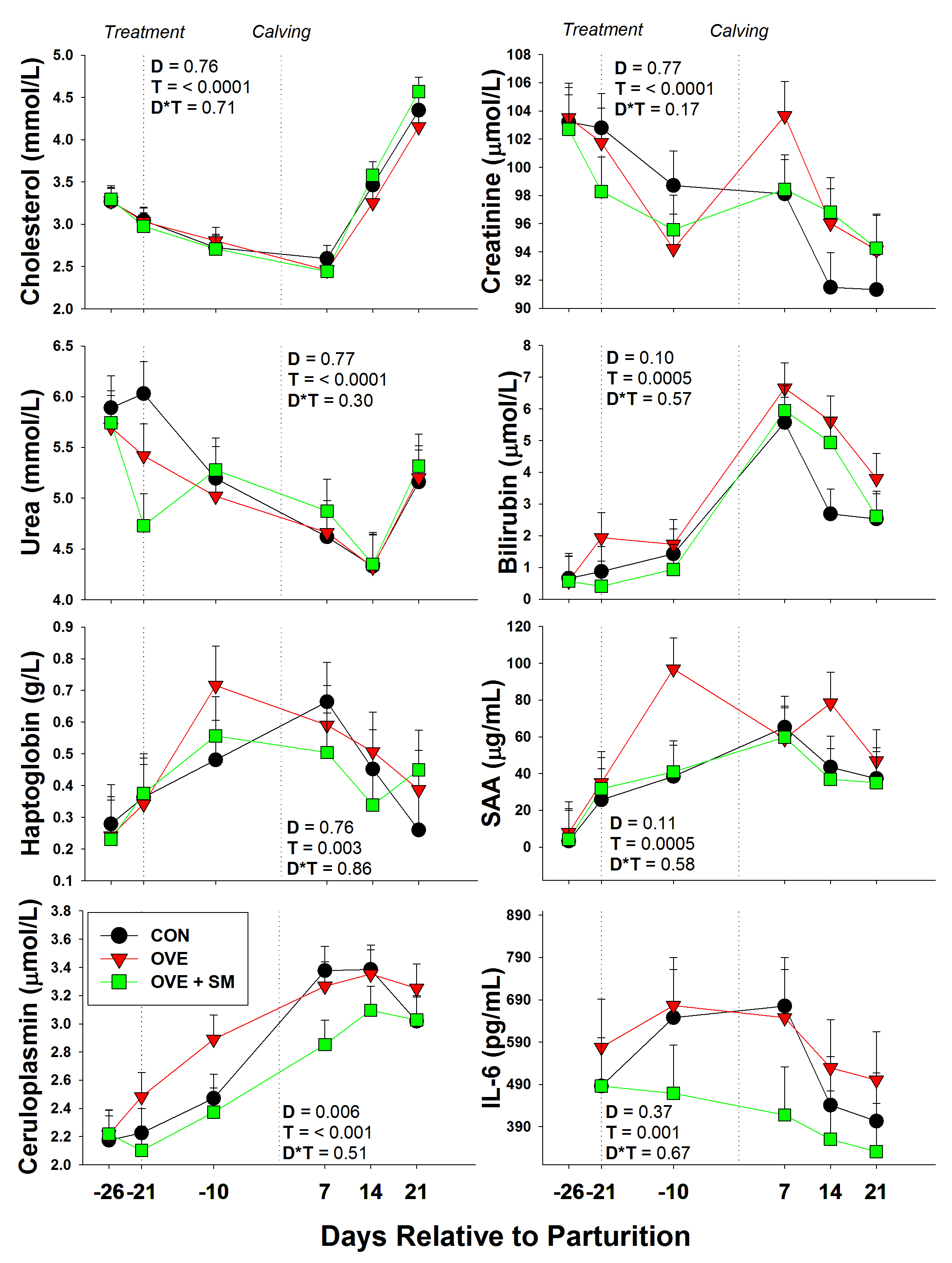


**Figure S2.** Effect of prepartum diet on biomarker of oxidative stress and antioxidant status in dairy cows during the transition period.

**
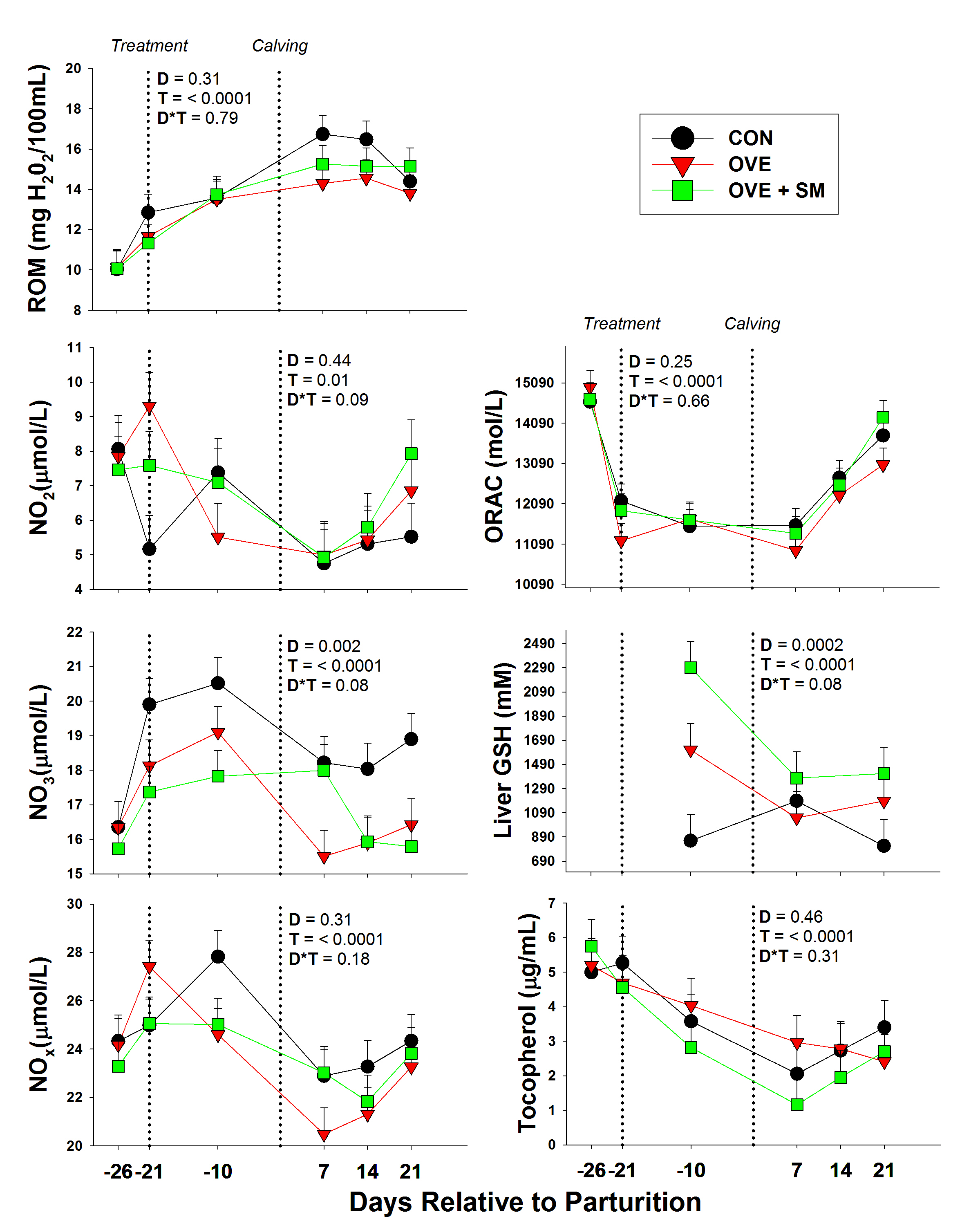
**

**Figure S3.** Effect of prepartum diet on hepatic expression (log_2_ back-transformed LSM) of the methionine cycle genes in dairy cows during the transition period.


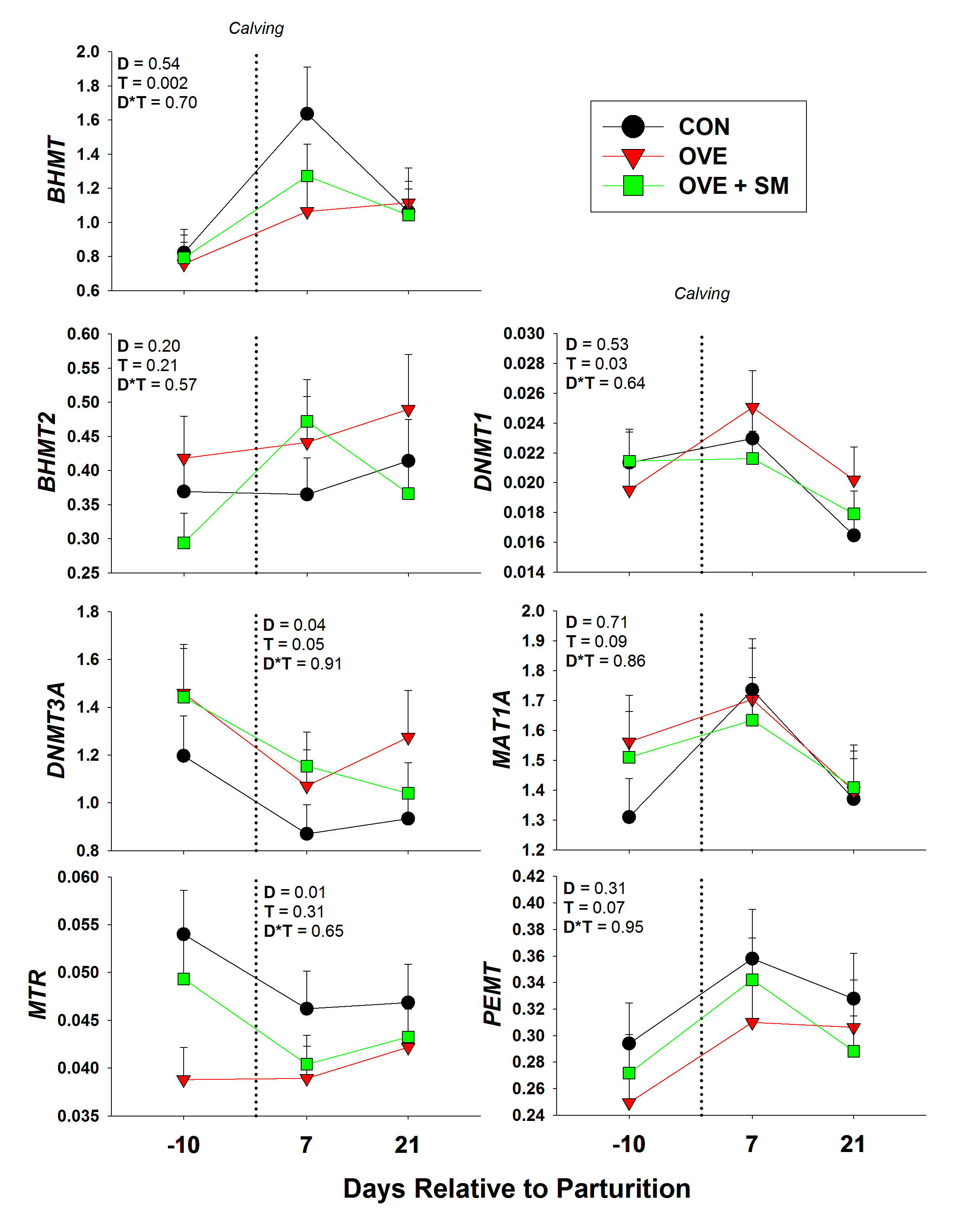


**Figure S4.** Effect of prepartum diet on hepatic expression (log_2_ back-transformed LSM) of genes involve in fatty acid metabolism and energy metabolism in dairy cows during the transition period.

**
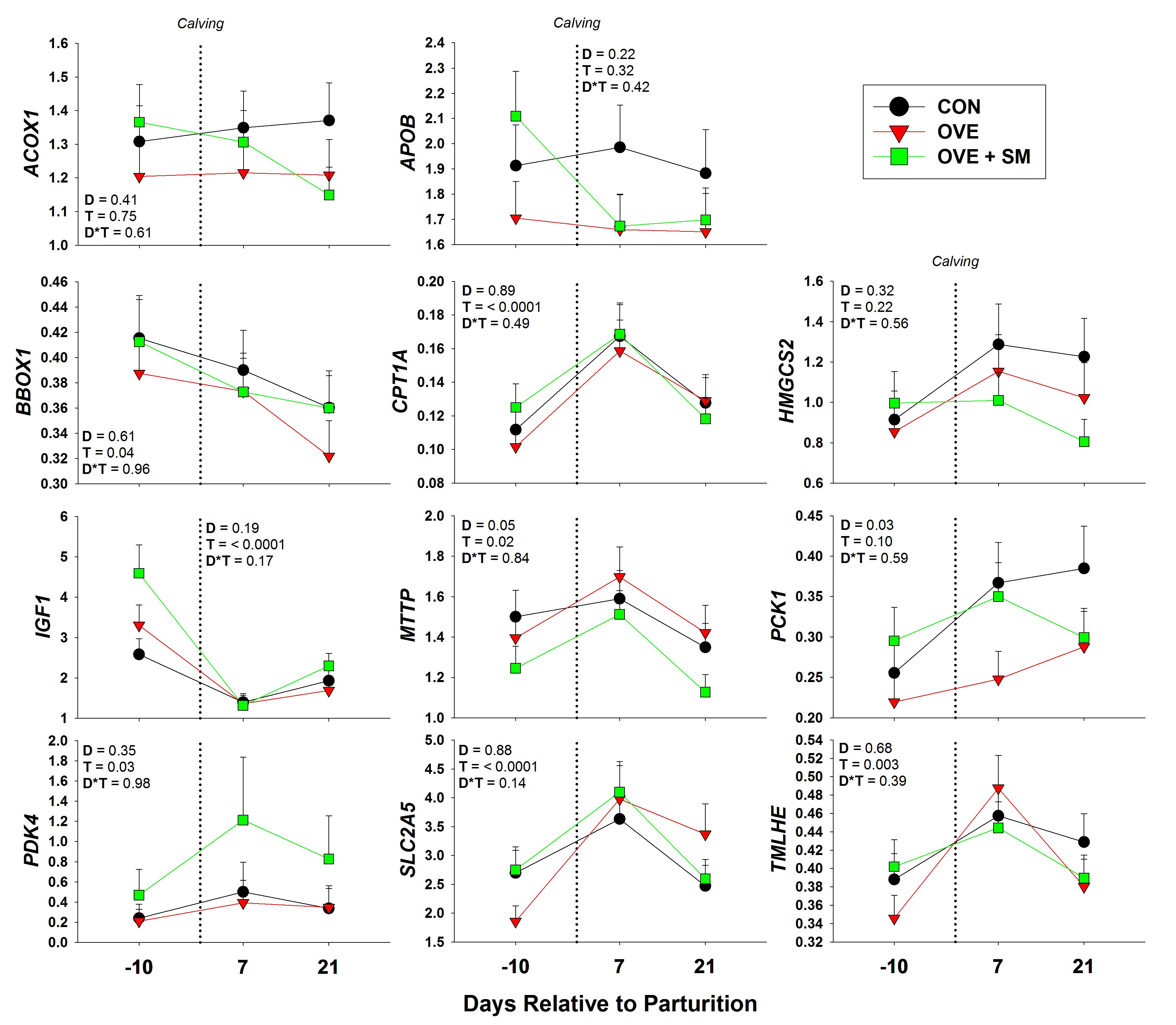
**

**Figure S5.** Effect of prepartum diet on hepatic expression (log_2_ back-transformed LSM) of genes involved in the antioxidant system in dairy cows during the transition period.

**
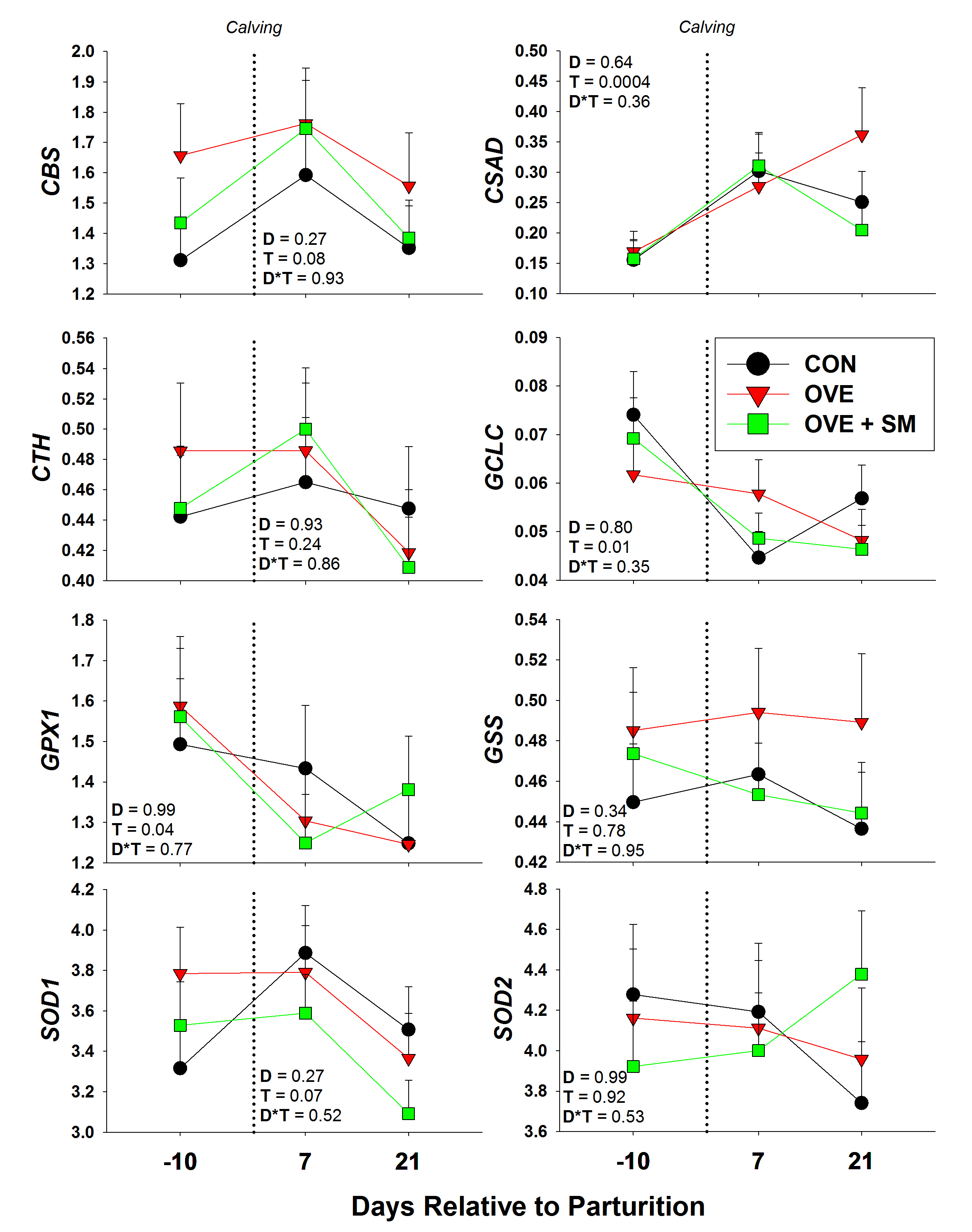
**

**REFERENCES**

1. Khan MJ, Jacometo CB, Graugnard DE, Correa MN, Schmitt E, Cardoso F, Loor JJ: **Overfeeding Dairy Cattle During Late-Pregnancy Alters Hepatic PPARalpha-Regulated Pathways Including Hepatokines: Impact on Metabolism and Peripheral Insulin Sensitivity**. *Gene Regul Syst Bio* 2014, **8**:97-111.

2. Vailati Riboni M, Meier S, Priest NV, Burke CR, Kay JK, McDougall S, Mitchell MD, Walker CG, Crookenden M, Heiser A *et al*: **Adipose and liver gene expression profiles in response to treatment with a nonsteroidal antiinflammatory drug after calving in grazing dairy cows**. *Journal of dairy science* 2015, **98**(5):3079-3085.
